# Perception of clinical research among patients and healthy volunteers of clinical trials

**DOI:** 10.1007/s00228-022-03366-3

**Published:** 2022-07-27

**Authors:** Felix Bergmann, Peter Matzneller, Maria Weber, Lusine Yeghiazaryan, Thorsten Fuereder, Thomas Weber, Markus Zeitlinger

**Affiliations:** 1 Department of Clinical Pharmacology, Medical University of Vienna, Währinger Gürtel 18-20, 1090 Vienna, Austria; 2 Clinical Division of Plastic and Reconstructive Surgery, Department of Surgery, Medical University of Vienna, Währinger Gürtel 18-20, 1090 Vienna, Austria; 3 Service of Rheumatology, Hospital of Merano (SABES-ASDAA), Via Rossini 5, 39012 Merano, Italy; 4 Center for Medical Statistics, Informatics, and Intelligent Systems, Medical University of Vienna, Währinger Gürtel 18-20, 1090 Vienna, Austria; 5 Division of Oncology, Department of Medicine I, Medical University of Vienna, Währinger Gürtel 18-20, 1090 Vienna, Austria; 6 Division of Gastroenterology and Hepatology, Department of Medicine III, Medical University of Vienna, Währinger Gürtel 18-20, 1090 Vienna, Austria

**Keywords:** Clinical research, Treating physicians, Volunteers

## Abstract

**Purpose:**

Clinical research relies on data from patients and volunteers, yet the target sample size is often not achieved. Here, we assessed the perception of clinical research among clinical trial participants to improve the recruitment process for future studies.

**Methods:**

We conducted a single-center descriptive and exploratory study of 300 current or former participants in various phase I–III clinical trials. Questionnaires were either distributed to current clinical trial participants or emailed to former subjects.

**Results:**

Subjects strongly agreed or agreed that contributing to improving medical care (> 81%), contributing to scientific research (> 79%), and trusting their treating physicians (> 77%) were motives for study participation. Among healthy volunteers, financial motives positively correlated with the number of clinical trials they had participated in (*p* < 0.05). Higher age positively correlated with expectation of best available treatment during study participation among patients (*p* < 0.05). Less than 8% of all subjects expressed “great concern” about the potential risks of sharing their personal information as part of the study. Subjects displayed “great trust” or “trust” in medical staff (86.6%) and in government research institutions (76.4%), and “very little trust” or “little trust” in pharmaceutical companies (35.4%) and health insurance companies (16.9%).

**Conclusion:**

Altruistic motives and trust in treating physicians were predominant motives for clinical trial participation. Older patients expected to receive the best available treatment during participation. Healthy volunteers who reported financial motives had participated in more clinical trials. Consistent with great trust in medical staff and government research institutions, little concern was expressed about the misuse of personal data during the trial.

**Supplementary Information:**

The online version contains supplementary material available at 10.1007/s00228-022-03366-3.

## Introduction

Unbiased clinical safety and efficacy assessments of novel therapeutic approaches largely rely on randomized controlled trials (RCTs) [1–3]. However, the inability to recruit and retain sufficient numbers of participants remains a common problem in achieving conclusive results [4–8]. This may result in underpowered clinical trials, in which clinically relevant differences are reported as statistically insignificant [9]. Thus, several studies have investigated influential factors of trial recruitment and participation. Key influential factors include altruistic motives [10–14], such as contribution to scientific research, financial incentives [13, 15–18], and the doctor-patient relationship [11, 19, 20].

Another aspect closely related to clinical research is the handling of personal data collected during clinical trials. The European Medicines Agency (EMA) argues that access to adequately anonymized data collected from clinical trials may benefit the process of drug development [21]. Several studies highlight the potential benefits of data sharing, such as the acceleration of scientific discovery, improvement of cost-effectiveness, enabling comparisons between different populations, improving surveillance of drug safety and efficacy, increasing sample size, improvement of comparator-effectiveness analysis, and reduction of duplicated efforts [21–23]. Although hesitancy and concern have been expressed about widespread data sharing [24, 25], a previous study demonstrated that most clinical trial participants believe that the potential benefits outweigh the risks [14].

Furthermore, certain personality traits have been correlated with motives for clinical trial participation [26]. The Big Five personality traits (BIFI), also known as the OCEAN model, use survey data to describe aspects of personality [27]. The theory proposes the following five dimensions to describe the human psyche: Openness, Conscientiousness, Extraversion, Agreeableness, and Neuroticism [28]. However, when conducting clinical trials, time is often limited, and personality may not be of primary interest. In this case, the Ten-Item Personality Inventory (TIPI) offers an abbreviated (2-measure) alternative, when brevity is of importance [29, 30].

In light of these findings, the aim of this study was to further assess clinical trial participants’ motives for trial participation and their views on the risks of data sharing and to investigate personality as a predictor of clinical trial participation behavior.

## Methods

### Trial design

We conducted a single-center descriptive and exploratory study at the General Hospital, Vienna, Austria.

### Participants

The patient collective of the study consisted of current or former participants of phase I–III clinical studies conducted at the Medical University of Vienna. All participants underwent an informed consent process and were enrolled between March 2019 and September 2020.

To generate a diverse sample of clinical trials, two identical adaptations of the questionnaire were created, one in physical print, the other as a digital survey. Printed surveys were distributed at 5 different departments to their clinical trial patients. Patients either completed the form immediately or returned it later by mail. The digital survey was distributed via email to patients or subjects who had either participated in a clinical trial in the past or were currently participating in one and had agreed to receive information about future trials.

### Questionnaire

A 50-item questionnaire was designed to capture key parameters reflecting participants’ views on the recruitment process, motives for participation, trust in fields of scientific research, and their opinions on clinical trial data sharing with respect to their most recent trial participation. It also included questions regarding the participant’s demographics and assessed personality traits based on the TIPI. The survey was designed under consultation of the Statistics Department of the Medical University of Vienna and underwent further refinement after a pilot phase with 5 participants. The full text of the study questionnaire can be found in the Supplementary Appendix.

### Ten-item personality inventory

The TIPI consists of 10 short personality questions [29]. It incorporates 2 questions to evaluate each trait of the OCEAN model. Answers were recorded on a Likert scale ranging from 1 to 5 (1 = Strongly disagree, 5 = Strongly agree). One of the corresponding questions in each case functions as a negative control and must be inverted (i.e., 1 is recoded to 5, 2 is recoded to 4). Finally, the mean of both answers correlating to their respective trait is assessed.

### Statistical analysis

Statistical analysis was performed using version 4.0.3 of the commercially available computer program R. Bar charts and frequency tables were created for each categorical variable. Spearman correlation coefficients were calculated between the variables. Correlation tests were also performed to determine significance. The significance level *α* was set at 0.05. All percentages respectively refer to the total number of responses obtained for each question.

## Results

### Study population

**Fig. 1:**
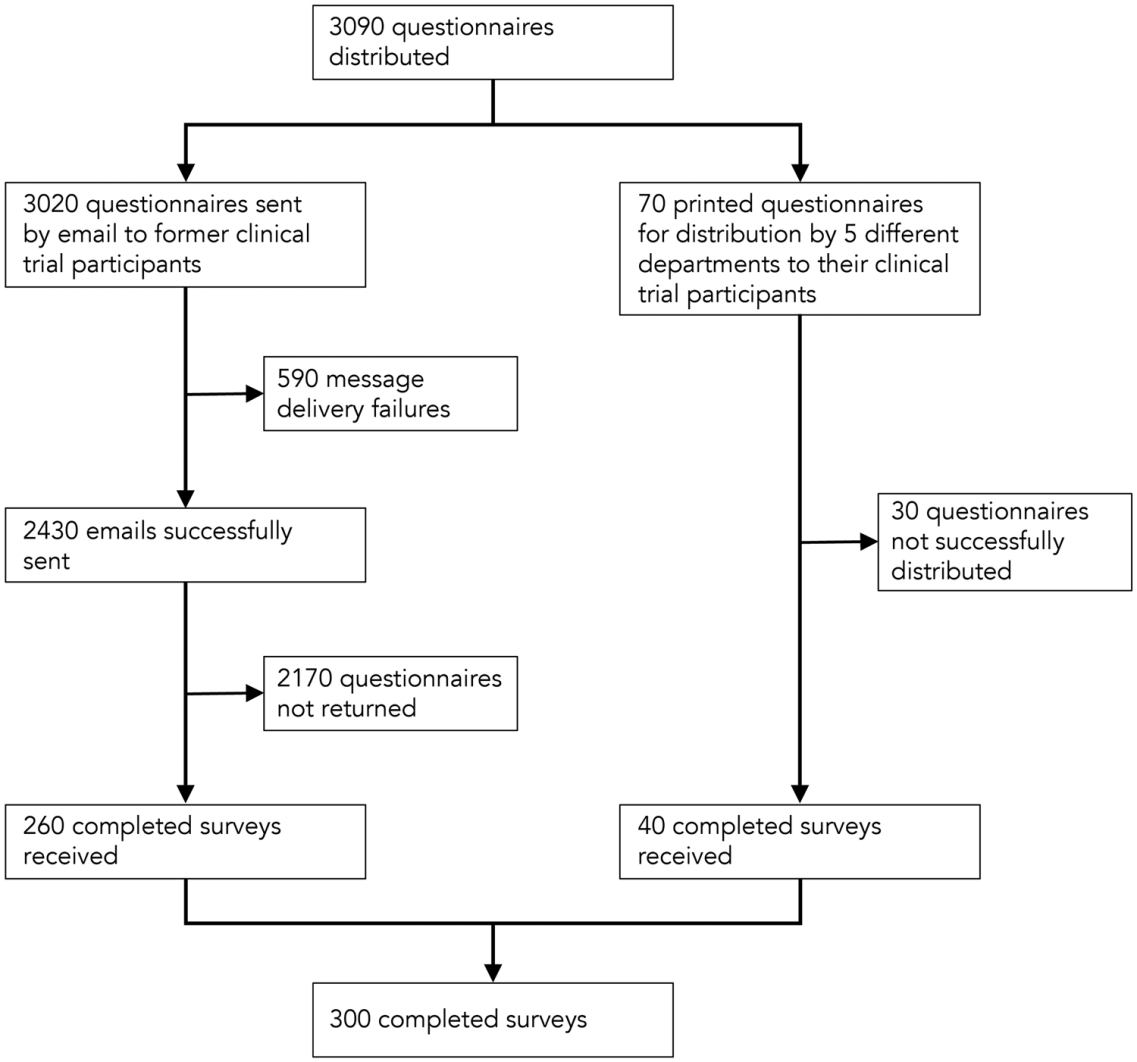
Flow chart of study participants

A total of 300 participants were included in the study (Fig. 1). Of the 3020 emails extracted from the database, 2430 emails were successfully sent (80.5%). Message delivery failures were received from the remaining 590 emails. Two hundred sixty completed surveys (10.7%) were returned from the 2430 invited trial participants. The number of emails successfully sent, yet not opened (e.g., marked as spam or unread) could not be determined. A total of 40 of the 70 printed questionnaires (57.1%) were returned. Table 1 describes the characteristics of the sample.

**Table 1:** Sample characteristics as reported in the survey

**Characteristic**	**No. of participants, no. (%) (*N* = 300)**
Female sex	165 (55.6)
Age	
≤ 30	70 (24.1)
31 to 59	189 (64.9)
≥ 60	32 (11)
Nationality	
Austria	228 (81.4)
Germany	23 (8.2)
Other	29 (10.4)
Participation as	
Patient	84 (28.3)
Healthy volunteer	213 (71.7)
Annual net income	
Less than €20,000	88 (32)
€21,000 to €40,000	129 (46.9)
€40,000 to €60,000	38 (13.8)
€61,000 to €80,000	12 (4.4)
€81,000 to €100,000	5 (1.8)
€100,000 or higher	3 (1.1)
Trial experience	
1 trial	157 (53.4)
2–5 trials	106 (36.1)
6 or more trials	31 (10.5)

### Process of informed consent

Our results show that most participants recalled undergoing an elaborate process of informed consent with a typical duration of 5–15 min (65.6%). Goals, execution, and time expenditure of the study were most often recalled (> 90%), whereas information regarding personal rights, data protection, and anonymity were often reported as missing (recalled by 59.9%, 63.9%, and 68%, respectively) (Fig. S1). The majority of participants were either approached by the study doctor (39.8%) or their attending physician (19.7%) and reported easily comprehensible study goals (86.9% very good comprehensibility or good comprehensibility) (Figs. S2 and S3). Subjects were most often contacted in person (38.4%), via telephone (34%), or email (19.4%) (Fig. S4).

### Motives

#### Patients

**Fig. 2:**
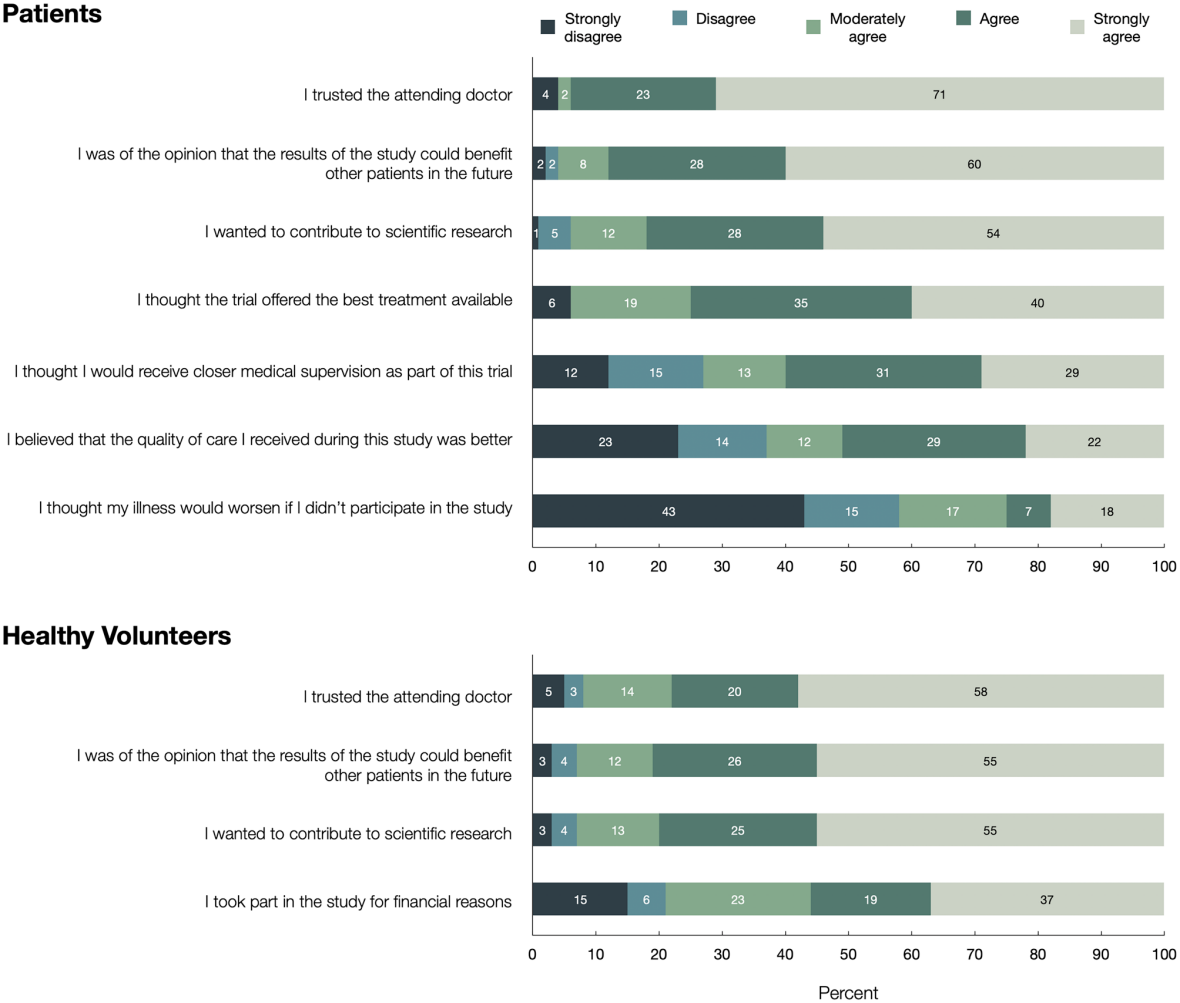
Motives for trial participation among patients and healthy volunteers. Shown is the response distribution to a question worded as, “What were your motives for participating in the study?”

Predominant motives for trial participation among patients were trust in the attending physician, improving future medical care, and contribution to research (94.1%, 86.9%, and 81.9% strongly agreed or agreed, respectively) (Fig. 2). The majority of patients also strongly agreed or agreed that they expected best possible medical treatment (74.7%), closer medical supervision (60.3%), and better quality of care (51.3%). Patients did not express substantial concern that their illness would worsen, if they did not participate in the trial (58.3% strongly disagreed or disagreed). Increasing age positively correlated with expecting the best available treatment (*p* < 0.05) (Fig. S5).

#### Healthy volunteers

56.3% of healthy volunteers strongly agreed or agreed to participate based on financial incentives, outweighed by altruistic motives such as contribution to improvement of medical care and scientific research (81.6% and 79.7%, respectively) and trust in their attending doctor (77.6%). However, only the indication of financial motives for participation positively correlated with the number of studies subjects had participated in (*p* < 0.05) (Fig. S6). Among healthy volunteers, female sex and increasing age positively correlated with the motives, “contribution to scientific research” (*p* < 0.05) and negatively correlated with financial motivation (*p* < 0.05). Increasing age also positively correlated with the motive “improvement of care for future patients” (*p* < 0.05). Higher income negatively correlated with financial incentives and the motive of trusting their attending doctor (*p* < 0.05).

#### Expected beneficiaries of clinical trials

**Fig. 3:**
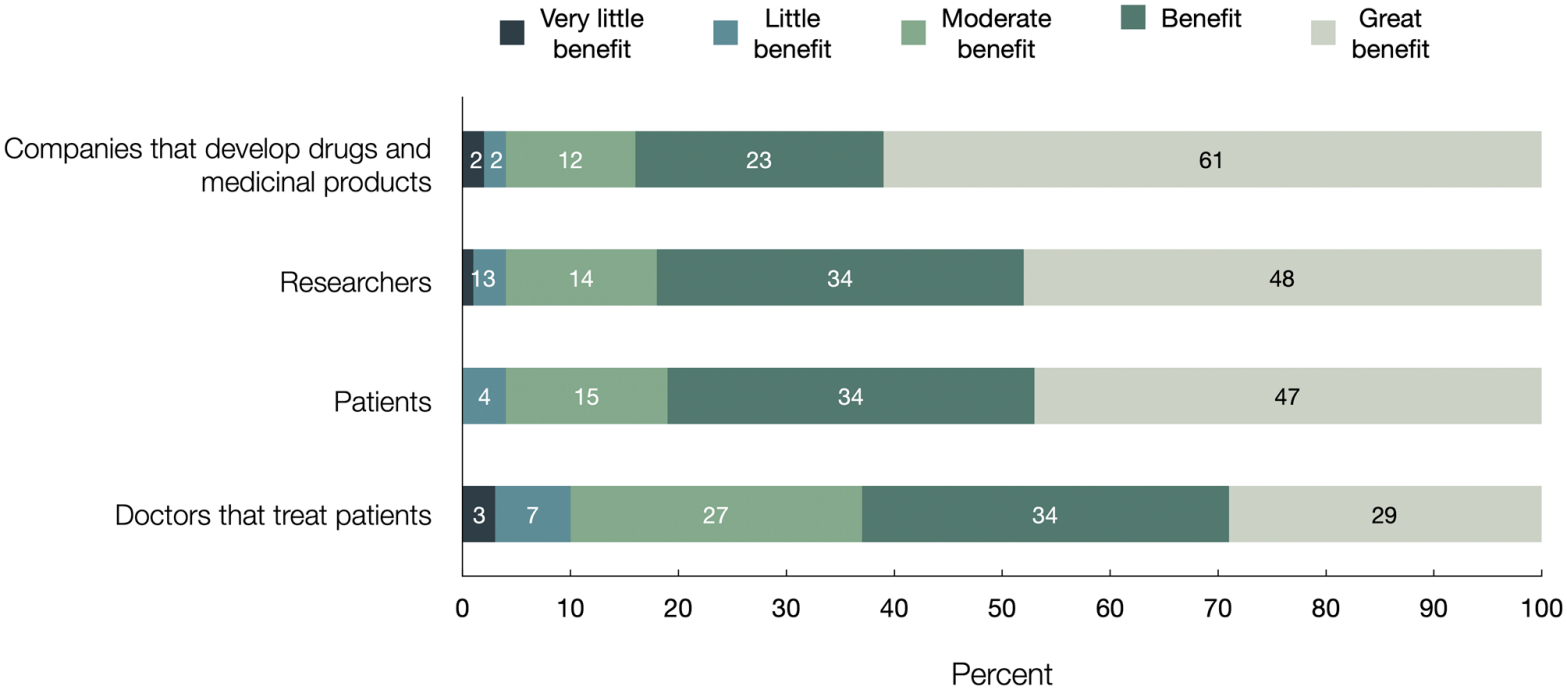
Expected beneficiaries of clinical trials. Shown is the response distribution to a question worded as, “How much do you think the following groups would benefit from clinical trials?”

More than 80% of all respondents expected companies that develop drugs and medicinal products (83.7%), researchers (82.1%), and patients (80.9%) to “greatly benefit” or “benefit” from clinical trials (Fig. 3). 63.1% of subjects had this expectation for doctors that treat patients.

### Data protection

**Fig. 4:**
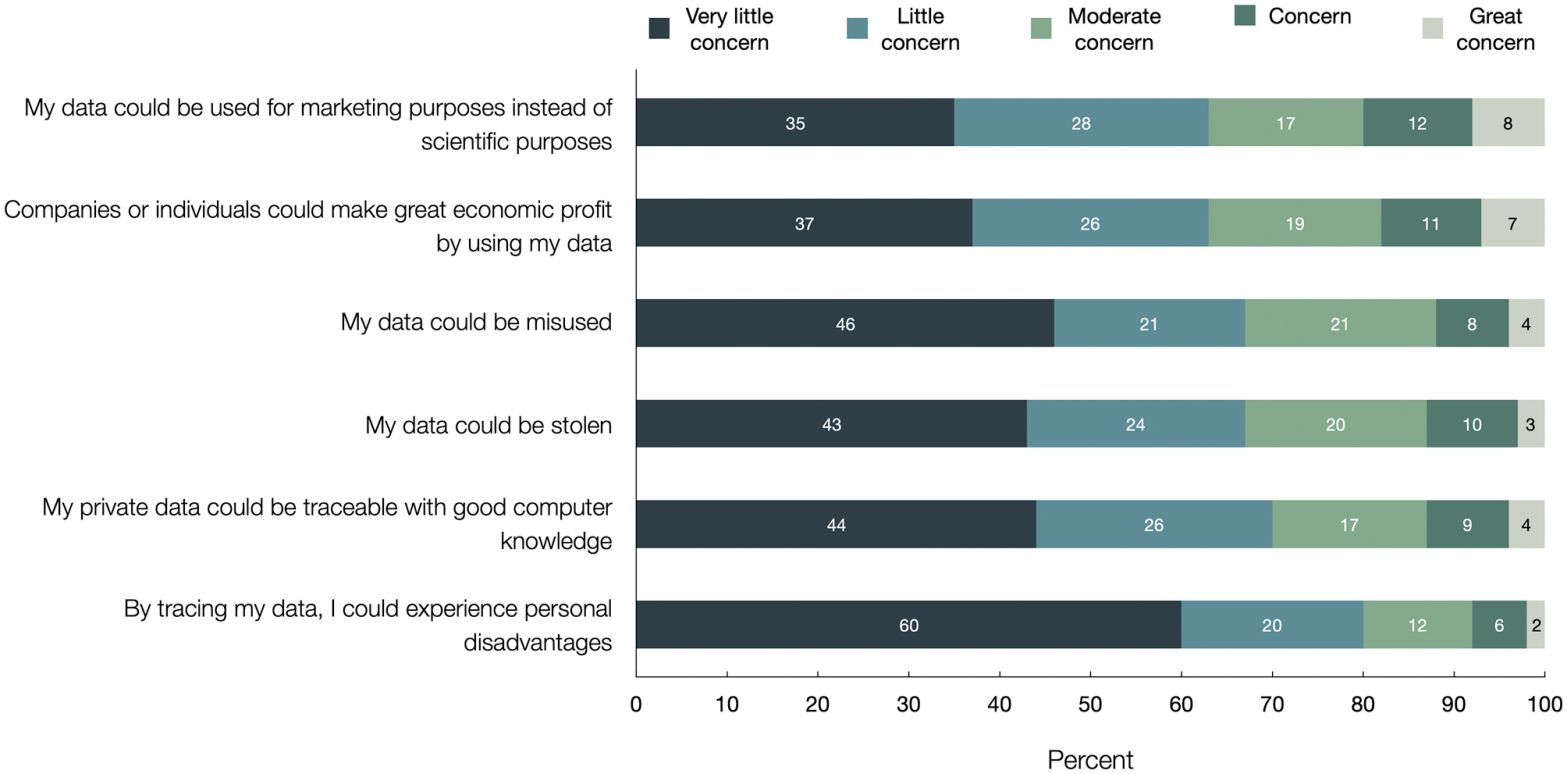
Concern about the potential consequences of sharing personal information. Shown is the response distribution to a question worded as, “During your participation in clinical trials, you have consented to the disclosure of your personal data to third parties in anonymized form. How concerned are you about the following possible consequences of sharing anonymous clinical trial data?”

Less than 10% of all respondents said they were “very concerned” and less than one-third were “concerned” or “moderately concerned” about the potential consequences of sharing personal data (Fig. 4). Respondents were most concerned that their data could be used for marketing purposes instead of scientific purposes (20% “very concerned” or “concerned”) or that companies or individuals could make a great economic profit by using their data (17.2%). Participants were least concerned that they could experience personal disadvantages (8.8% “very concerned” or “concerned”) or that their data could be traceable with good computer knowledge (12.8%). Greater trust in scientific research correlated with lower concern regarding data sharing (Fig. S7). No disparity was observed when comparing healthy volunteers and patients, independent of gender and age (*p* > 0.05, data not shown).

### Trust in fields of scientific research

**Fig. 5:**
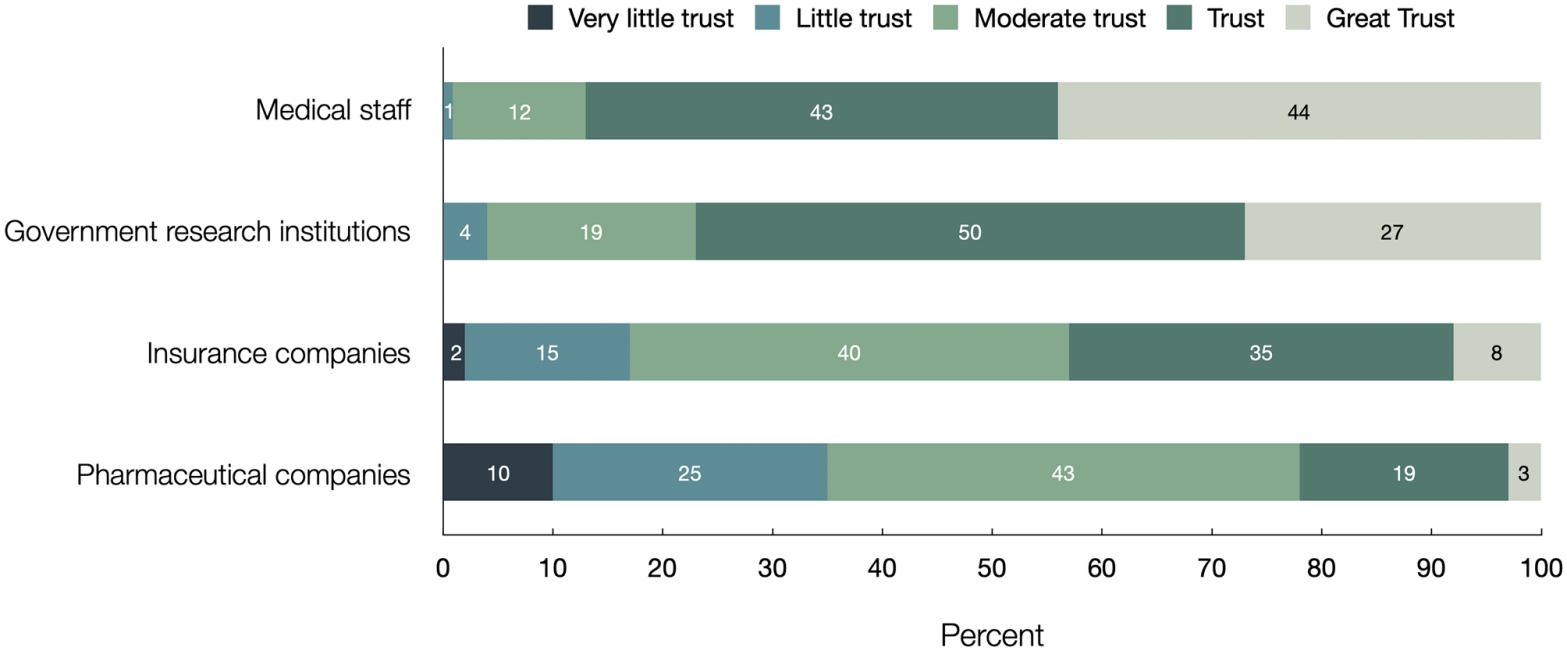
Trust in fields of scientific research. Shown is the response distribution to a question worded as, “How great is your trust in...”

Figure 5 documents participants’ levels of trust in fields of scientific research. Strong majorities of respondents expressed “great trust” or “trust” in medical staff (86.6%) and in government research institutions (76.4%); however, they showed “very little trust” or “little trust” in pharmaceutical companies (35.5%) and health insurance companies (16.9%). Trust in pharmaceutical companies, was higher among patients than healthy volunteers (*p* < 0.05) and increased with higher age among all individuals (*p* < 0.05).

### Ten-item personality index

All pairs of questions that corresponded to a personality trait correlated indirectly (*p* < 0 0.05), with the exception of Agreeableness (*p* = 0.265) (Figure S8). The following mean scores were calculated for patients and healthy volunteers, respectively: Openness: 3.6_patients_, 3.6_healthy volunteers_; Conscientiousness: 4.0_patients_, 3.8_healthy volunteers_; Extraversion: 3.5_patients_, 3.5_healthy volunteers_; Agreeableness: 3.5_patients_, 3.3_healthy volunteers_; Neuroticism: 2.6_patients_, 2.5_healthy volunteers_. Patients showed higher Agreeableness than healthy volunteers (*p* < 0.05). Higher age corresponded with higher levels of Conscientiousness. Female sex was directly proportional to Neuroticism (p < 0.05) and Conscientiousness (*p* < 0.05). Figures S9–11 summarize correlations between TIPI scores with motives for participation, views on data protection and trust in fields of scientific research. Although some significances were calculated, no significant trend could be detected.

## Discussion

The present study assessed the perception of clinical research among patients and healthy volunteers of clinical trials in a European cohort. While most previous studies have focused on a single aspect of clinical trials in either patients or healthy volunteers, this work provides a comprehensive overview of how both subject groups perceive various areas of clinical research.

Consistent with other literature, the predominant motives for participation among our subjects were altruistic motives and trust in attending doctors [10–14, 19, 20]. Over 80% of all participants strongly agreed or agreed that contribution to scientific research, improvement of medical care for future patients, and great trust in their treating physician were motives for their participation in clinical trials. In addition, the majority (> 80%) of all respondents expected researchers, patients, and companies that develop drugs and medicinal products to profit from clinical trials.

According to Good Clinical Practice (GCP) standard, the participation of patients in clinical trials is generally not remunerated. Rather, the opportunity to receive a new treatment alone represents the trial’s benefit and has been documented to be an influential factor for patients [12]. However, our data suggest that health benefits were not primary motivators among the included patients, and fear of disease progression was not of substantial concern. Rather, these motives were outweighed by altruistic considerations and trust in attending physicians. Nevertheless, with age, patients did show increasing interest in receiving the best available treatment. These findings underscore the importance of the physician–patient relationship during recruitment and the informed consent process, as well as the potential value of highlighting the public benefits derived from the study and the fact that participation in the study will not compromise standard of care when recruiting older patients.

In contrast, it is widely accepted to include monetary incentives in the recruitment of healthy subjects, which are often a necessary component for the successful completion of clinical trials [18, 31]. Furthermore, there is no evidence suggesting that commonly used payments represent undue remuneration or that financial inducements may distort healthy volunteers’ risk perception associated with the clinical trial [32, 33]. Yet surprisingly, only 56% of healthy volunteers strongly agreed or agreed to have participated due to financial benefits. Thus, although financial compensation is often the focus of recruitment, it may not be the only incentive for subjects to participate in clinical trials. Instead, similarly to patients, the most dominant motives in healthy volunteers were altruistic motives and trust in their attending physicians. Conversely, yet in line with literature, healthy volunteers that indicated financial motives for participation had participated in more clinical trials [34]. This suggests that remuneration may indeed be linked to repeated clinical trial participation in healthy volunteers. Finally, female sex and older age positively correlated with altruistic motives and negatively correlated with financial incentives.

As part of their participation in clinical trials, participants consented to the disclosure of data to third parties in anonymized form. Contrary to public hesitancy [24, 25], subjects did not express considerable concern about the potential risks of data sharing. The perceived risk was lower than that found in previous studies [14, 35]; less than 10% of participants were “very concerned” about any risks mentioned in the questionnaire. The greatest concern was expressed about the misuse of personal data for marketing purposes instead of scientific purposes (8.5% “very concerned”) or for capitalization by companies or individuals (6.4%). A recent systematic review highlights the importance of communication during the process of informed consent and suggests that trust in scientific research and clinicians may mitigate concern regarding data sharing [36]. In agreement with these findings, our subjects expressed considerable trust (76.4% show “great trust” or “trust”) in government research institutions and in medical staff (86.6%), which correlated with lower concern about the consequences of data sharing. Similarly, most participants reported an elaborate, thorough and easily comprehensible process of informed consent. However, we acknowledge that clinical trial participants may represent those, who are least concerned about the potential violations of data protection and most enthusiastic about contributing to scientific progress. Thus, the perception and concerns of data sharing may differ from those of the general public or patients.

When examining personality traits, there was a significant difference between patients and healthy volunteers only in the trait of Agreeableness, with lower scores documented in the healthy volunteers. Although some sporadic significances were calculated when comparing personality traits with motives for participation, views on data sharing and trust in fields or research, no significant trend could be detected. Therefore, the TIPI did not function as an accurate predictor of clinical trial participation behavior. However, this may in part be due the loss of content validity and reliability when using abbreviated measures such as the TIPI [30].

## Limitations

Most participants took part in phase I clinical trials, thus resulting in a large number of young, healthy individuals. This resulted in an underrepresentation of old (> 60 years of age) participants and an imbalance between healthy volunteers (71.7%) and patients (28.3%). Furthermore, the sample consisted exclusively of individuals that have participated in clinical trials. Therefore, it was not possible to compare our findings to a control group, i.e., subjects that have declined participation in the past, or to determine factors responsible for non-participation. Hence, it was also not possible to determine whether the motives that were strongly represented in our sample were decisive for participation in clinical trials or whether these motives were only shared by individuals who generally have an affinity for trial participation. Despite the substantial size of our sample, response rates were low, possibly leading to nonresponse bias. In addition, we acknowledge the bias of selective memory. We were unable to determine the date and time of initial participation; hence, participation may lie far in the past and some data may have been recalled erroneously. Finally, no adjustment was conducted for multiple testing.

## Conclusion

In our study, altruistic motives and trust in attending physicians were the predominant motives for clinical trial participation in healthy volunteers and patients. This underscores the importance of the physician–patient relationship during the informed consent process. In addition, with age, patients increasingly expected to receive the best available treatment by participating in trials. Healthy volunteers who reported financial motives for their participation had participated in more clinical trials. Therefore, it may be beneficial to emphasize these benefits during the process of informed consent.

Our subjects expressed little concern about the potential risks of sharing their personal information as part of their participation. Finally, in our study, the Ten-Item Personality Index did not function as an accurate predictor of clinical trial behavior.

## Supplementary Information

Below is the link to the electronic supplementary material.

Supplementary file1 (DOCX 2493 KB)

## Data Availability

The data that support the findings of this study are available from the corresponding author upon reasonable request.

## References

[1] Odgaard‐Jensen J, Vist GE, Timmer A, Kunz R, Akl EA, Schünemann H, Briel M, Nordmann AJ, Pregno S, Oxman AD (2011) Randomisation to protect against selection bias in healthcare trials Cochrane. Database Syst Rev 4. 10.1002/14651858.MR000012.pub3 10.1002/14651858.MR000012.pub3 PMC7150228 21491415

[2] Eichler HG, Pignatti F, Schwarzer-Daum B (2021). Randomized controlled trials versus real world evidence: neither magic nor myth. Clin Pharmacol Ther.

[3] Collins R, Bowman L, Landray M, Peto R (2020). The magic of randomization versus the myth of real-world evidence. N Engl J Med.

[4] Fogel DB (2018). Factors associated with clinical trials that fail and opportunities for improving the likelihood of success: a review. Contemp Clin Trials Commun.

[5] Kadam RA, Borde SU, Madas SA (2016). Challenges in recruitment and retention of clinical trial subjects. Perspect Clin Res.

[6] McDonald AM, Knight RC, Campbell MK et al (2006) What influences recruitment to randomised controlled trials? A review of trials funded by two UK funding agencies. Trials 7:9. 10.1186/1745-6215-7-9 10.1186/1745-6215-7-9 PMC1475627 16603070

[7] Newington L, Metcalfe A (2014). Factors influencing recruitment to research: qualitative study of the experiences and perceptions of research teams. BMC Med Res Methodol.

[8] Ford JG, Howerton MW, Lai GY (2008). Barriers to recruiting underrepresented populations to cancer clinical trials: a systematic review. Cancer.

[9] Treweek S, Lockhart P, Pitkethly M, Cook JA, Kjeldstrøm M, Johansen M, Taskila TK, Sullivan FM, Wilson S, Jackson C, Jones R (2013) Methods to improve recruitment to randomised controlled trials: Cochrane systematic review and meta-analysis. BMJ Open 3. 10.1136/bmjopen-2012-002360 10.1136/bmjopen-2012-002360 PMC3586125 23396504

[10] Truong TH, Weeks JC, Cook EF, Joffe S (2011). Altruism among participants in cancer clinical trials. Clin Trials.

[11] Jenkins V, Fallowfield L (2000). Reasons for accepting or declining to participate in randomized clinical trials for cancer therapy. Br J Cancer.

[12] Godskesen T, Hansson MG, Nygren P (2015). Hope for a cure and altruism are the main motives behind participation in phase 3 clinical cancer trials. Eur J Cancer Care.

[13] Stunkel L, Grady C (2011). More than the money: a review of the literature examining healthy volunteer motivations. Contemp Clin Trials.

[14] Mello MM, Lieou V, Goodman SN (2018). Clinical trial participants’ views of the risks and benefits of data sharing. N Engl J Med.

[15] Martinson BC, Lazovich DA, Lando HA (2000). Effectiveness of monetary incentives for recruiting adolescents to an intervention trial to reduce smoking. Prev Med.

[16] Jia P, Furuya-Kanamori L, Qin Z-S et al (2020) Association between response rates and monetary incentives in sample study: a systematic review and meta-analysis. Postgrad Med J 97:501-510. 10.1136/postgradmedj-2020-137868 10.1136/postgradmedj-2020-137868 32848082

[17] Almeida L, Azevedo B, Nunes T (2007). Why healthy subjects volunteer for phase I studies and how they perceive their participation?. Eur J Clin Pharmacol.

[18] Tishler CL, Bartholomae S (2002). The recruitment of normal healthy volunteers: a review of the literature on the use of financial incentives. J Clin Pharmacol.

[19] Sood A, Prasad K, Chhatwani L (2009). Patients’ attitudes and preferences about participation and recruitment strategies in clinical trials. Mayo Clin Proc.

[20] Grant CH, Cissna KN, Rosenfeld LB (2000). Patients’ perceptions of physicians communication and outcomes of the accrual to trial process. Health Commun.

[21] Eichler H-G, Pétavy F, Pignatti F, Rasi G (2013). Access to patient-level trial data — a boon to drug developers. N Engl J Med.

[22] Sampson MG, Kang HM (2019) The path to open data. Nat Rev Nephrol 15:521. 10.1038/s41581-019-0188-6 10.1038/s41581-019-0188-6 31427809

[23] Mello MM, Francer JK, Wilenzick M et al (2013) Preparing for responsible sharing of clinical trial data. N Engl J Med 369:1651–1658. 10.1056/NEJMhle1309073 10.1056/NEJMhle1309073 24144394

[24] Papoutsi C, Reed JE, Marston C (2015). Patient and public views about the security and privacy of electronic health records (EHRs) in the UK: results from a mixed methods study Healthcare Information Systems. BMC Med Inform Decis Mak.

[25] Garrison NA, Sathe NA, Antommaria AHM (2016). A systematic literature review of individuals’ perspectives on broad consent and data sharing in the United States. Genet Med.

[26] Johnson MO (2000). Personality correlates of HIV vaccine trial participation. Personality Individ Differ.

[27] Goldberg LR (1990). Personality processes and individual differences - an alternative “Description of Personality”: the Big-Five factor structure. J Pers Soc Psychol.

[28] Goldberg LR (1993). The structure of phenotypic personality traits: author’s reactions to the six comments. Am Psychol.

[29] Gosling SD, Rentfrow PJ, Swann WB (2003). A very brief measure of the Big-Five personality domains. J Res Pers.

[30] Credé M, Harms P, Niehorster S, Gaye-Valentine A (2012). An evaluation of the consequences of using short measures of the Big Five personality traits. J Pers Soc Psychol.

[31] Grady C (2005). Payment of clinical research subjects. J Clin Investig.

[32] Bentley JP, Thacker PG (2004). The influence of risk and monetary payment on the research participation decision making process. J Med Ethics.

[33] Halpern SD, Karlawish JHT, Casarett D (2004). Empirical assessment of whether moderate payments are undue or unjust inducements for participation in clinical trials. Arch Intern Med.

[34] Fisher JA, McManus L, Wood MM (2018). Healthy volunteers’ perceptions of the benefits of their participation in phase I clinical trials. J Empir Res Hum Res Ethics.

[35] Whiddett R, Hunter I, Engelbrecht J, Handy J (2006). Patients’ attitudes towards sharing their health information. Int J Med Informatics.

[36] Hutchings E, Loomes M, Butow P, Boyle FM (2021). A systematic literature review of attitudes towards secondary use and sharing of health administrative and clinical trial data: a focus on consent. Syst Rev.

