# Supplementary material for: Perception of clinical research among patients and healthy volunteers of clinical trials
Source: Eur J Clin Pharmacol. 2022 Jul 27;78(10):1647–55. doi: 10.1007/s00228-022-03366-3 (PMC9482583; doi:10.1007/s00228-022-03366-3)
Supplement: Supplementary file 1 — Supplementary file1 (DOCX 2493 KB) [file 228_2022_3366_MOESM1_ESM.docx]

**Supplementary Appendix to:**

**Perception of Clinical Research Among Patients and Healthy Volunteers of Clinical Trials**

**European Journal of Clinical Pharmacology**

**Authors**

Felix Bergmann^1,2^, Peter Matzneller^1,3^, Maria Weber^1^, Lusine Yeghiazaryan^4^, Thorsten Fuereder^5^, Thomas Weber^6^, Markus Zeitlinger^1,*^

*Corresponding author: Markus.zeitlinger@meduniwien.ac.at

**Affiliations**

^1^ Medical University of Vienna, Department of Clinical Pharmacology, Währinger Gürtel 18-20, 1090 Vienna, Austria

^2^ Medical University of Vienna, Clinical Division of Plastic and Reconstructive Surgery, Department of Surgery, Währinger Gürtel 18-20, 1090 Vienna, Austria.

^3^ Service of Rheumatology, Hospital of Merano (SABES-ASDAA), Via Rossini 5, 39012 Merano, Italy

^4^ Medical University of Vienna, Center for Medical Statistics, Informatics, and Intelligent Systems, Währinger Gürtel 18-20, 1090 Vienna, Austria.

^5^ Medical University of Vienna, Department of Medicine I, Division of Oncology, Währinger Gürtel 18-20, 1090 Vienna, Austria

^6^ Medical University of Vienna, Department of Medicine III, Division of Gastroenterology and Hepatology, Währinger Gürtel 18-20, 1090 Vienna, Austria

**Table of Contents**

[Fig. S1 Recalled contents of the process of informed consent 3](#_Toc103102532)

[Fig. S2 First trial contact 3](#_Toc103102533)

[Fig. S3 Comprehensibility of study goals 4](#_Toc103102534)

[Fig. S4 Method of first contact 4](#_Toc103102535)

[Fig. S5 Association between expecting the best treatment available and age among patients 5](#_Toc103102536)

[Fig. S6 Association between the value placed on financial incentives and the study experience among healthy volunteers 5](#_Toc103102537)

[Fig. S7 Correlation between trust in fields of scientific research and concern regarding data sharing 6](#_Toc103102538)

[Fig. S8 Correlation between pairs of questions corresponding to respective personality traits 7](#_Toc103102539)

[Fig S9 Correlation between TIPI scores and gender, age, participation as a healthy volunteer, concern regarding data sharing and trust in fields of scientific research among all participants 7](#_Toc103102540)

[Fig. S10 Correlation between TIPI scores and motives for participation among healthy volunteers 8](#_Toc103102541)

[Fig. S11Correlation between TIPI scores and motives for participation among patients 9](#_Toc103102542)

[Full text of study questionnaire* 10](#_Toc103102543)


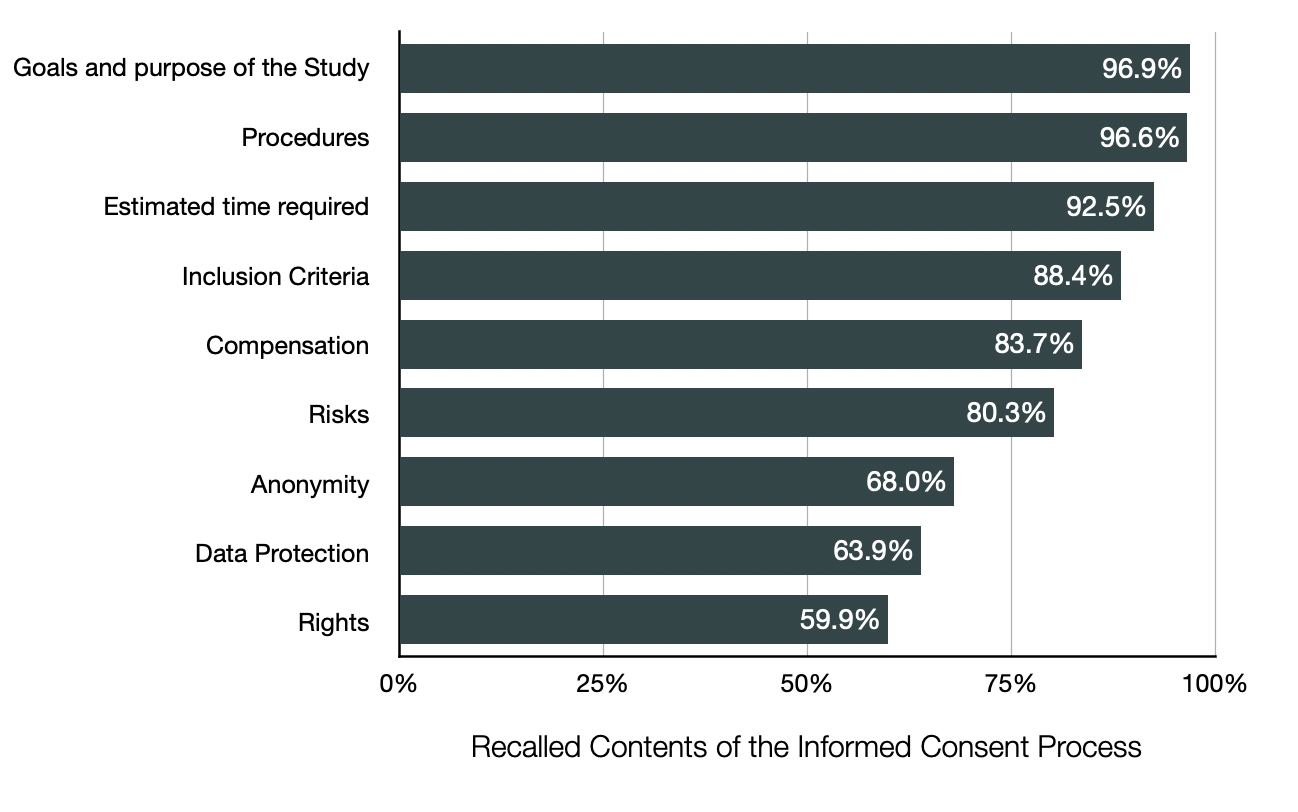


### Fig. S1 Recalled contents of the process of informed consent


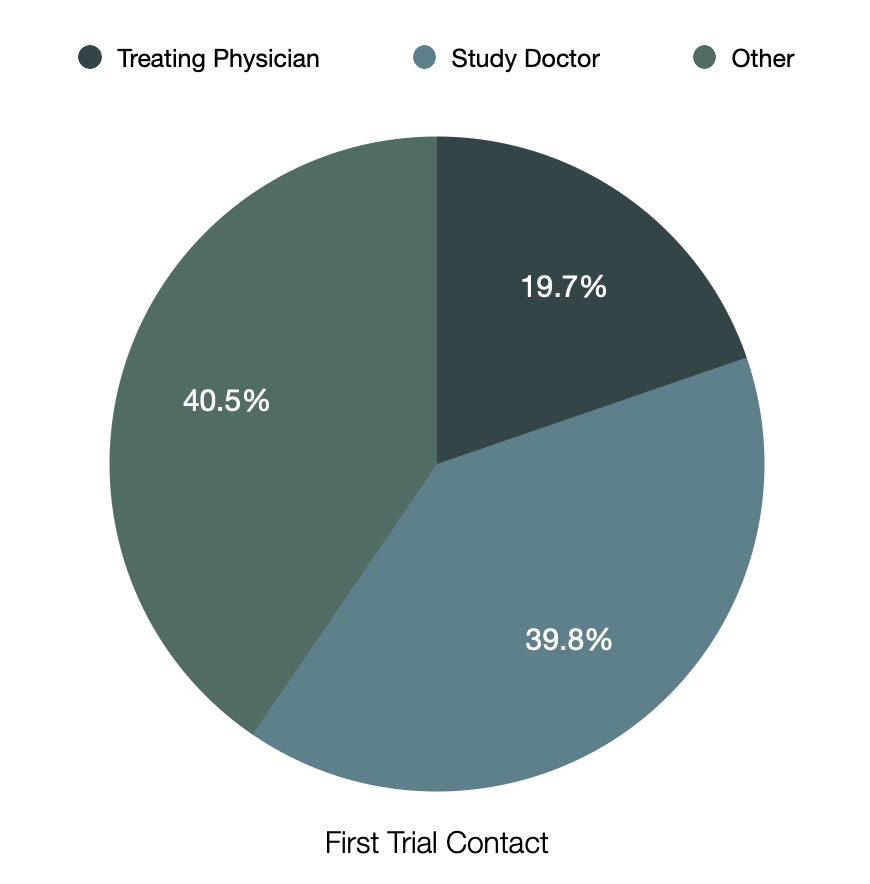


### Fig. S2 First trial contact


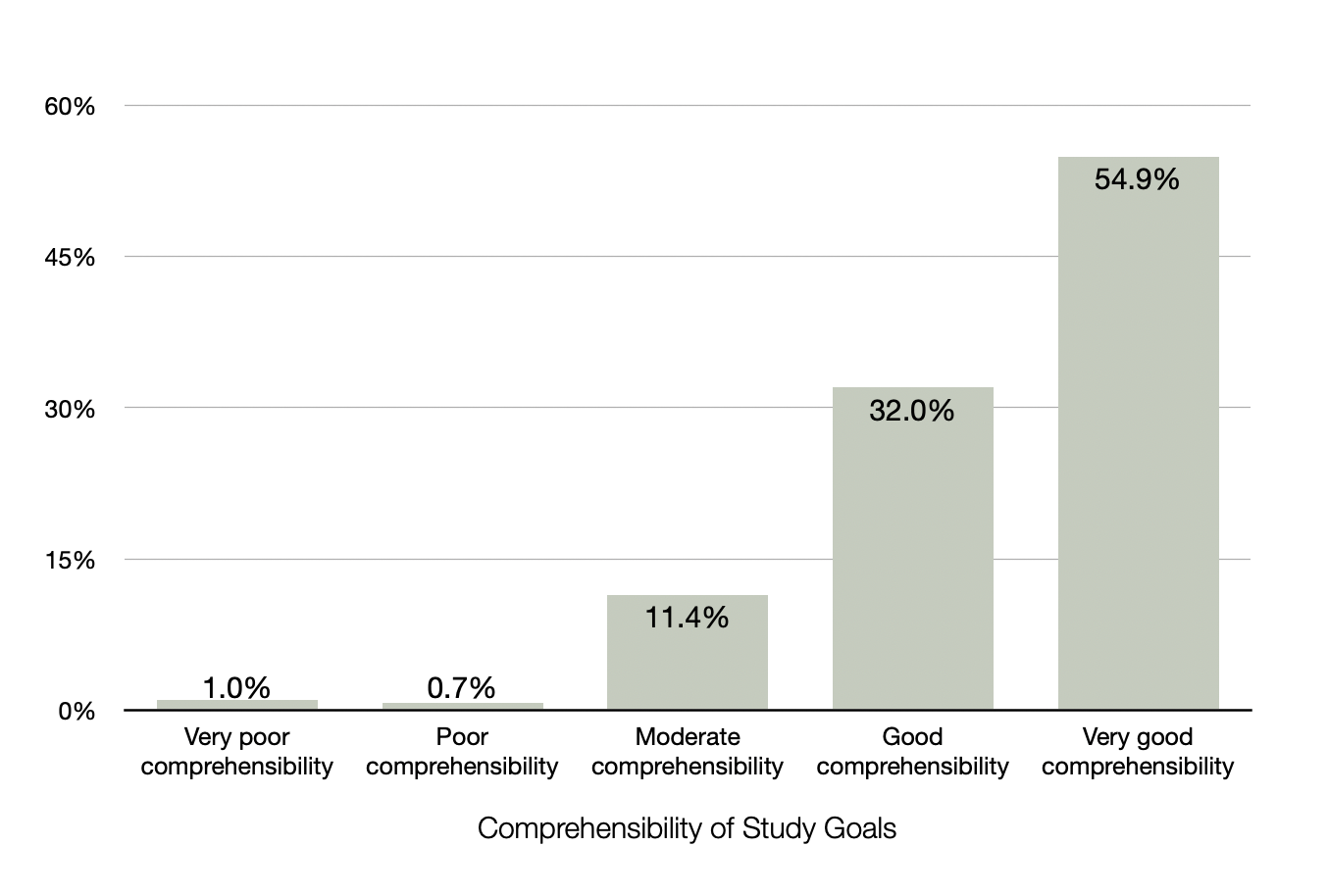


### Fig. S3 Comprehensibility of study goals


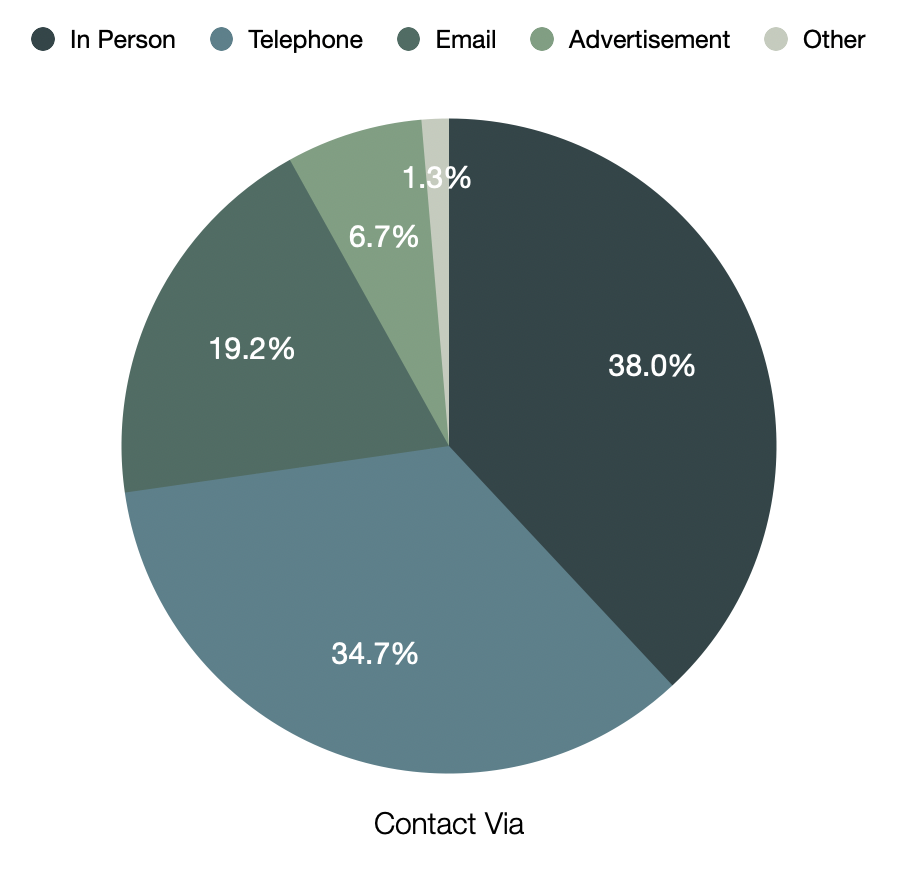


### Fig. S4 Method of first contact


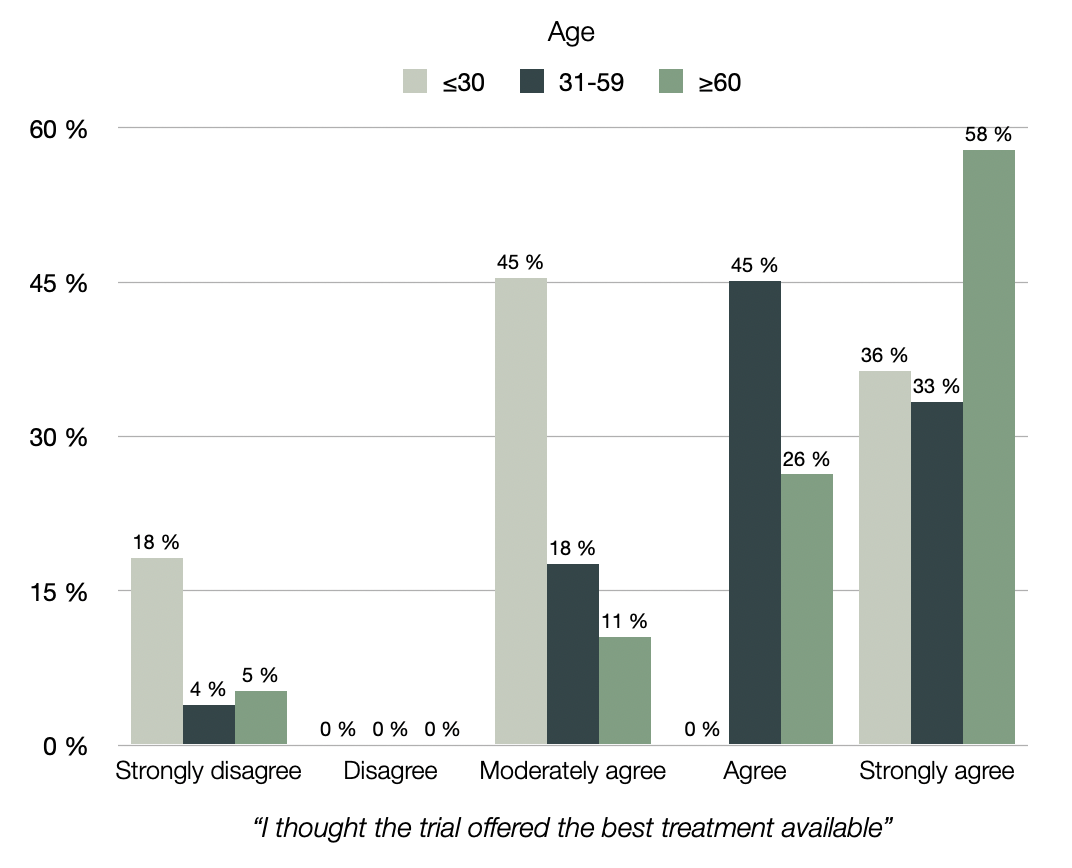


### Fig. S5 Association between expecting the best treatment available and age among patients


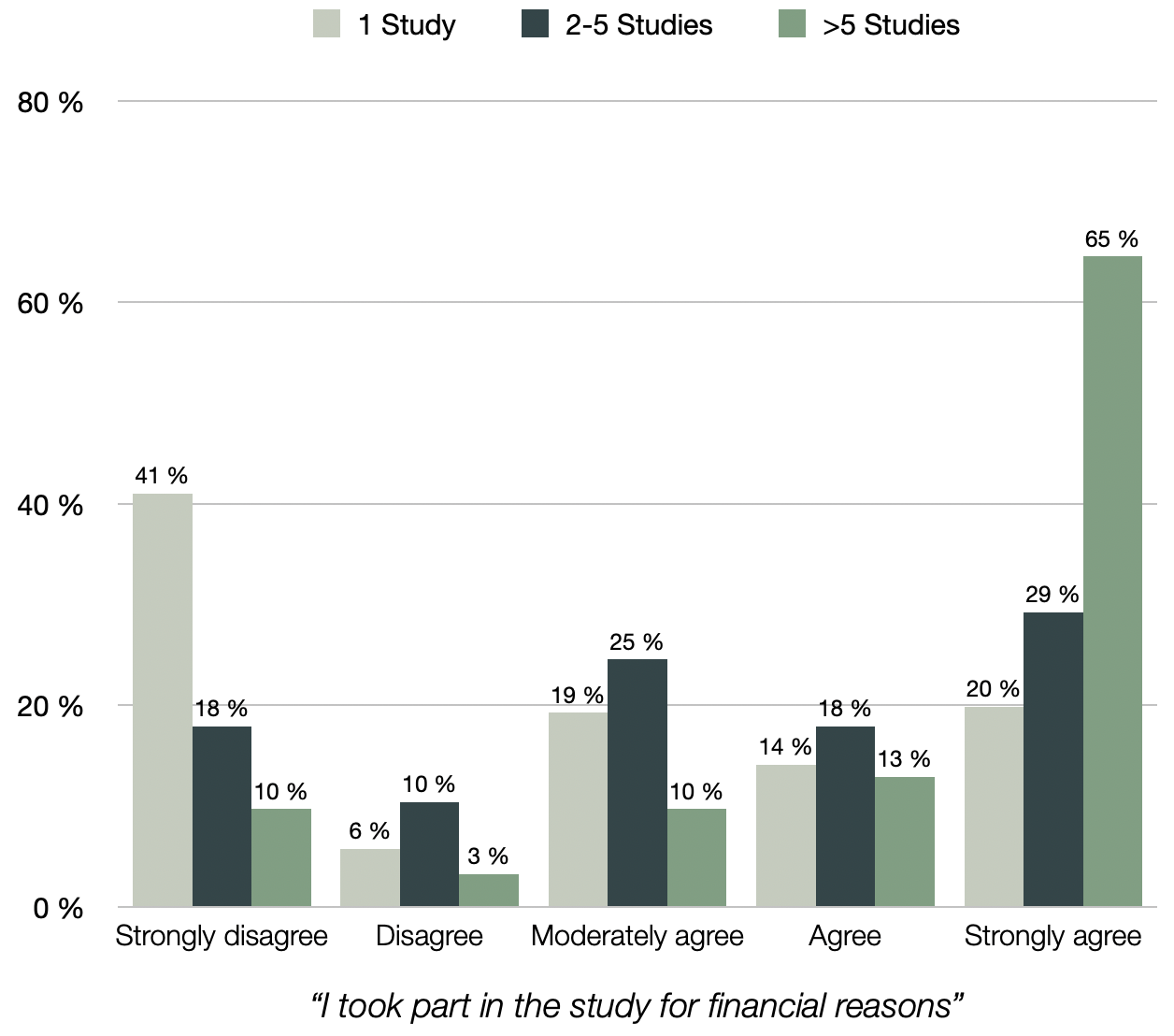


### Fig. S6 Association between the value placed on financial incentives and the study experience among healthy volunteers


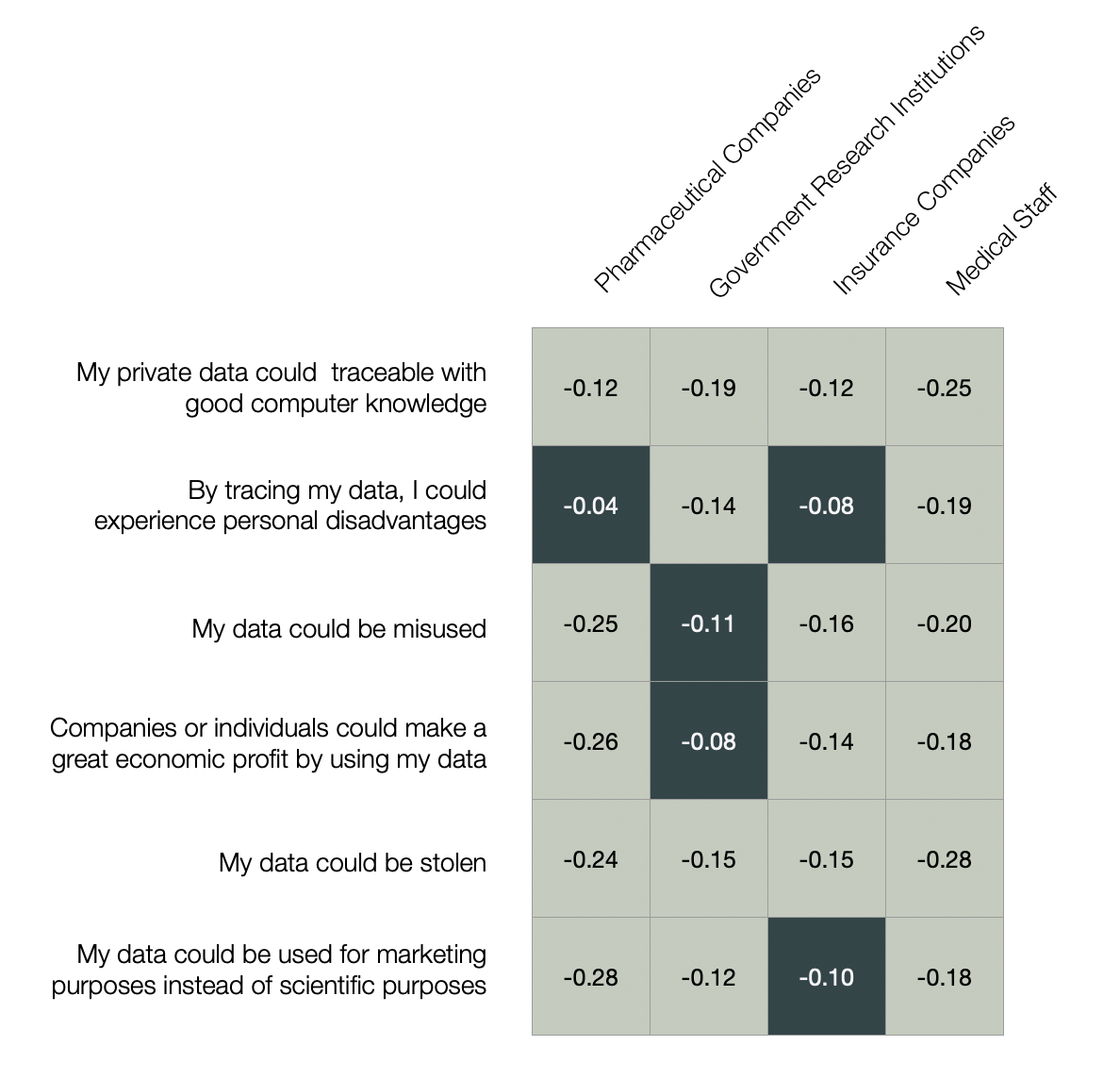


### Fig. S7 Correlation between trust in fields of scientific research and concern regarding data sharing

Shown are Spearman’s correlation coefficients. In addition, light shaded areas represent statistical significance (p<0.05).


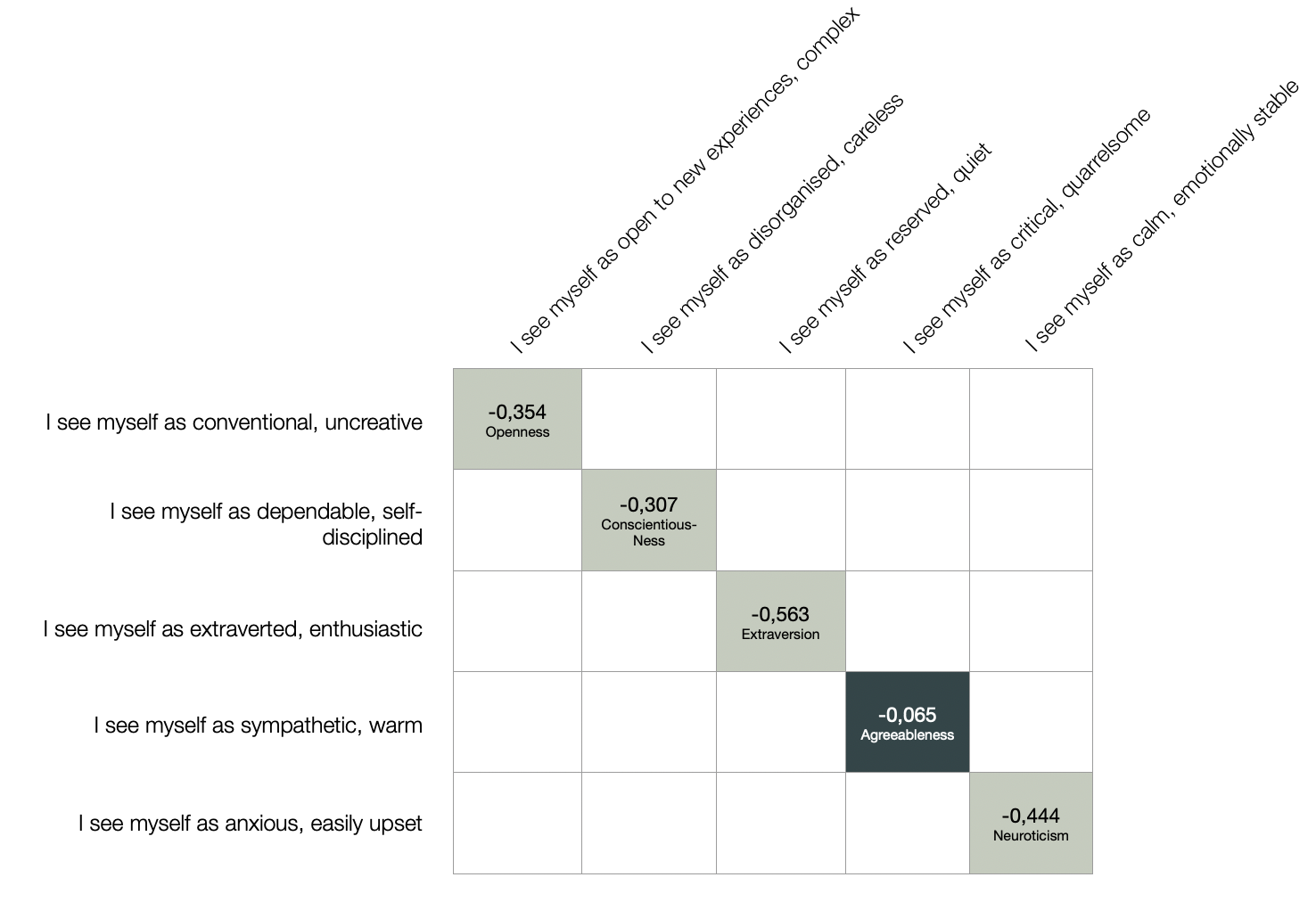


### Fig. S8 Correlation between pairs of questions corresponding to respective personality traits

Shown are Spearman’s correlation coefficients. In addition, light shaded areas represent statistical significance (p<0.05).


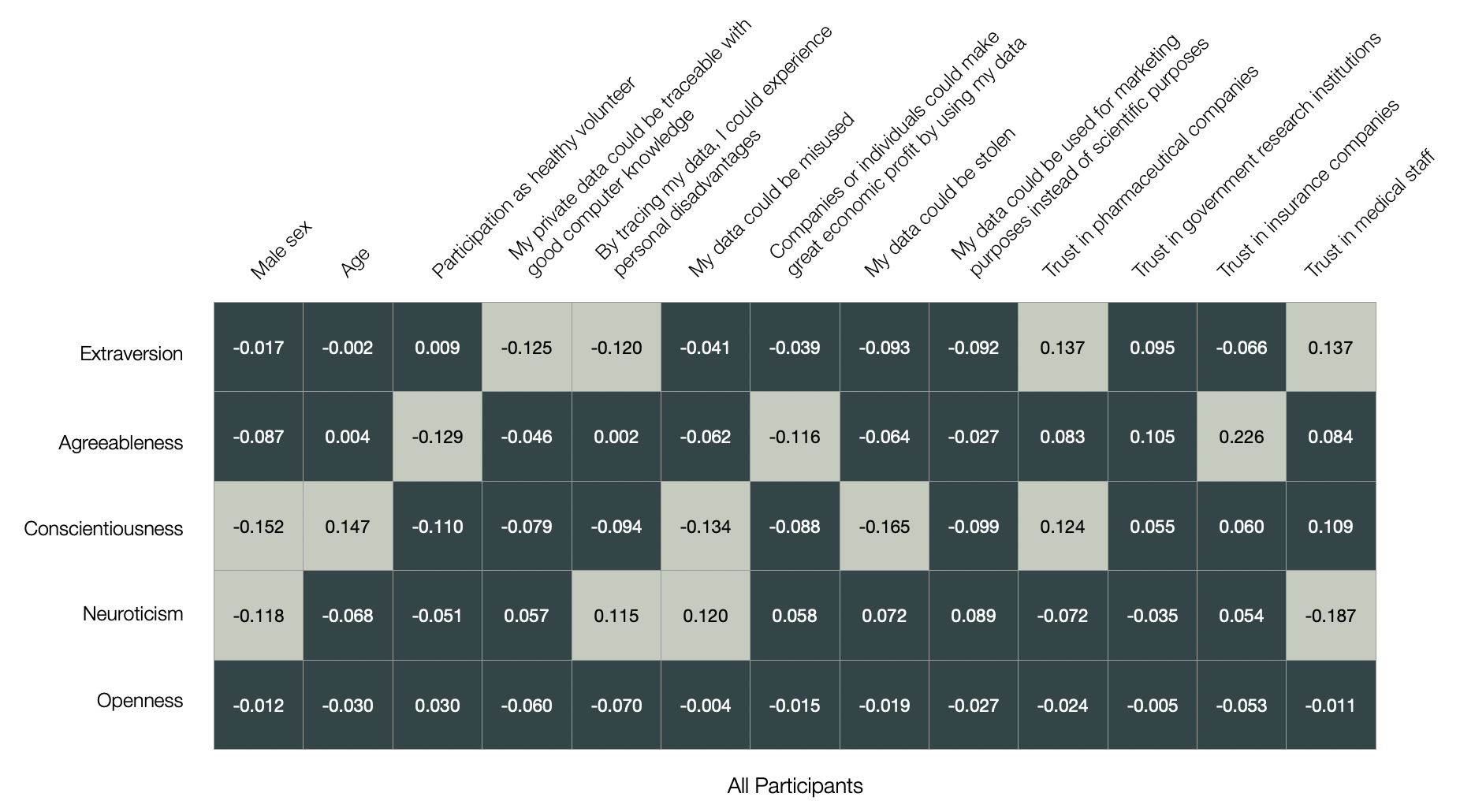


### Fig S9 Correlation between TIPI scores and gender, age, participation as a healthy volunteer, concern regarding data sharing and trust in fields of scientific research among all participants

Shown are Spearman’s correlation coefficients. In addition, light shaded areas represent statistical significance (p<0.05).


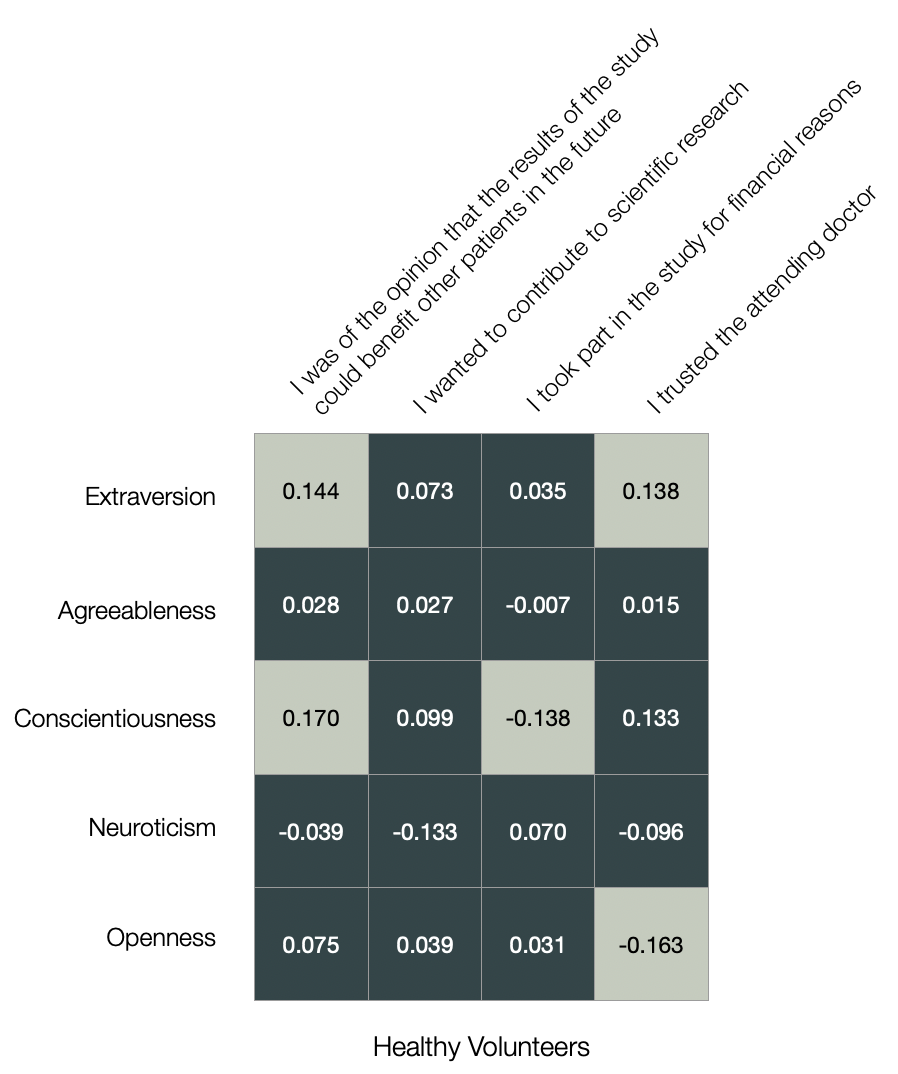


### Fig. S10 Correlation between TIPI scores and motives for participation among healthy volunteers

Shown are Spearman’s correlation coefficients. In addition, light shaded areas represent statistical significance (p<0.05).


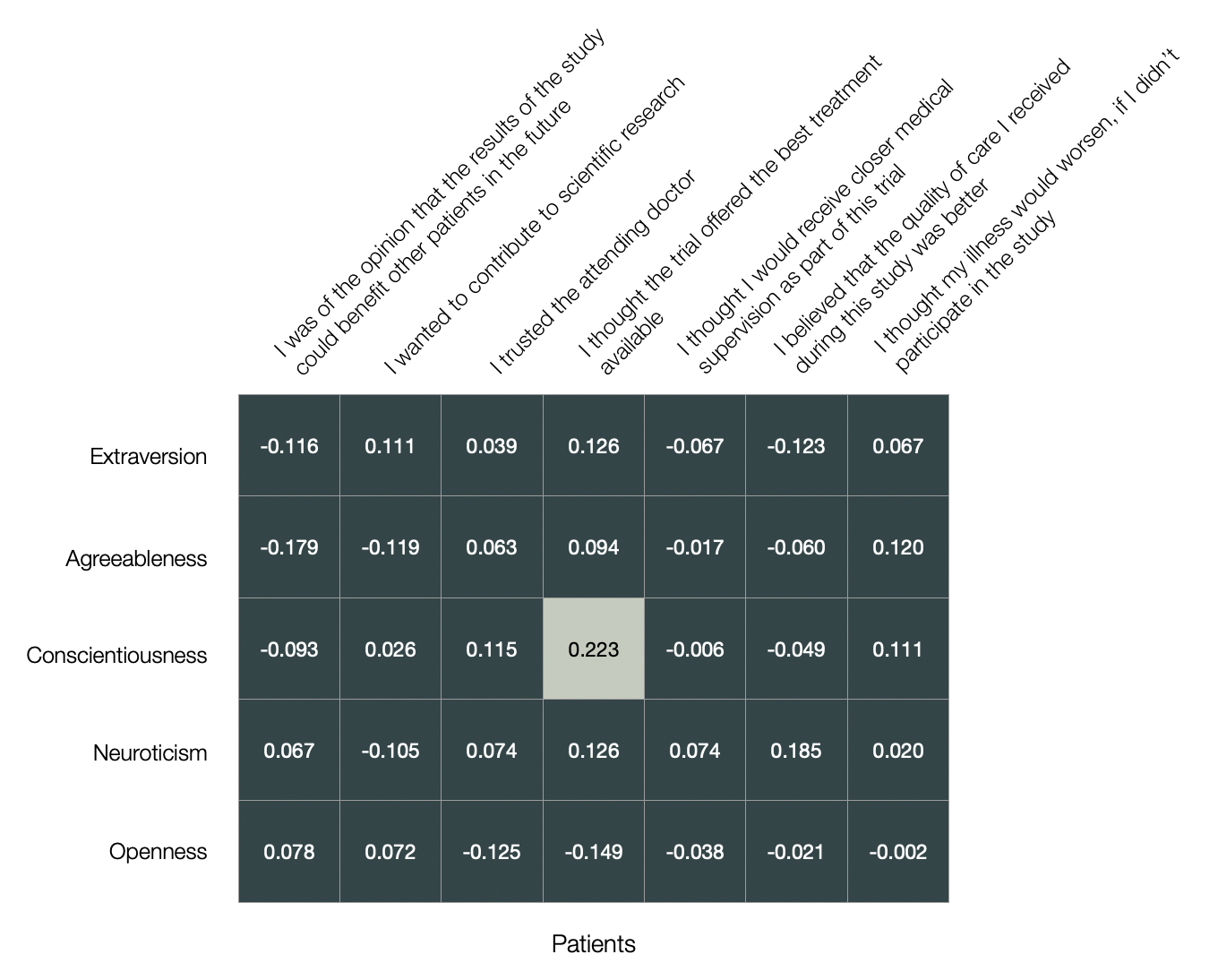


### Fig. S11Correlation between TIPI scores and motives for participation among patients

Shown are Spearman’s correlation coefficients. In addition, light shaded areas represent statistical significance (p<0.05).

### Full text of study questionnaire*

*Translated from german to english

**Perception of Clinical Research Among Clinical Trial Participants**

**What…**

You are invited to participate in a survey. We would like to ask people who have participated in clinical trials about their experiences.

This includes their views on the recruitment process, their hopes and fears, and their views on the data sharing that takes place as part of clinical trials.

We are interested in your opinion because you have experience as a clinical trial participant or are a parent of a participant. Your participation in this survey will not affect what happens to your own clinical trial data.

The survey is completely anonymous, and no data will be collected that allows conclusions to be drawn about the person completing it.

**How…**

If you choose to participate, you will take a 5- to 10-minute survey that asks your opinion about the benefits and risks of participating in a clinical trial, your motivations for participating, and the overall recruitment process. You can skip any questions you do not want to answer or stop the survey at any time.

Important: If you have already participated in several studies, the following questions refer only to the study in which you last participated.

**General Information**

**1) Who took part in the clinical trial?**

◻ Myself

◻ A relative: (son/mother...) my:____________________________

**I participated as a…**

◻ Healthy Volunteer

◻ Patient

**The following disease was the subject of the study**:

_____________________________________________________

I also suffer from the following diseases (name up to 3):

1) ____________________________________________________

2) ____________________________________________________

3) ____________________________________________________

**2) In which study and study phase (if known) did you last participate?**

______________________________________________________________

**3) How many studies have you or your relative participated in?**

◻ One Study

◻ 2-5 Studies

◻ More than 5 studies

**Study Experience**

**1) Contact was established by…**

◻ Treating physician

◻ Study Doctor

◻ Unknown

◻ ___________________________________________________________________

**2) Contact was established via…**

◻ Telephone

◻ Post

◻ Email

◻ In person

◻ Advertisement

◻ ___________________________________________________________________

**3) I was informed about the following during the informed consent process** (Multiple answers are possible)

◻ Goals and purpose of the study ◻ Compensation

◻ Inclusion criteria ◻ Data protection

◻ Procedure ◻ Anonymity

◻ Estimated time required ◻ Rights

◻ Risks

**4) My initial response after the informed consent process…**

◻ I was interested

◻ I was not interested

◻ I was overwhelmed

◻ I felt pressured by the study staff

**5) The goals of the study were…**

◻ Very comprehensible

◻ Comprehensible

◻ Moderately comprehensible

◻ Poorly comprehensible

◻ Very poorly comprehensible

**5) The duration of the informed consent process was…**

◻ < 5 Minutes

◻ 5-10 Minutes

◻ 11-15 Minutes

◻ 16-20 Minutes

◻ >20 Minutes

**Motives**

1. **What were your motives for participating in the study?**

|  | Strongly disagree | Disagree | Moderately agree | Agree | Strongly agree |
| --- | --- | --- | --- | --- | --- |
| I was of the opinion that the results of the study could benefit other patients in the future | ◻ | ◻ | ◻ | ◻ | ◻ |
| I wanted to contribute to scientific research | ◻ | ◻ | ◻ | ◻ | ◻ |
| I trusted the attending doctor | ◻ | ◻ | ◻ | ◻ | ◻ |
| I thought the trial offered the best treatment available | ◻ | ◻ | ◻ | ◻ | ◻ |
| I thought I would receive closer medical supervision as part of this trial | ◻ | ◻ | ◻ | ◻ | ◻ |
| I believed that the quality of care I received during this study was better | ◻ | ◻ | ◻ | ◻ | ◻ |
| I thought my illness would worsen if I didn’t participate in the study | ◻ | ◻ | ◻ | ◻ | ◻ |
| I took part in the study for financial reasons | ◻ | ◻ | ◻ | ◻ | ◻ |
|  |  |  |  |  |  |

**2) How much do you think the following groups would benefit from clinical trials?**

|  | Very little benefit | Little benefit | Moderate benefit | Benefit | Great benefit |
| --- | --- | --- | --- | --- | --- |
| Companies that develop drugs and medicinal products | ◻ | ◻ | ◻ | ◻ | ◻ |
| Researchers | ◻ | ◻ | ◻ | ◻ | ◻ |
| Patients | ◻ | ◻ | ◻ | ◻ | ◻ |
| Doctors that treat patients | ◻ | ◻ | ◻ | ◻ | ◻ |

**3) How great is your trust in…**

|  | Very little  trust | Little trust | Moderate trust | Trust | Great trust |
| --- | --- | --- | --- | --- | --- |
| Medical staff | ◻ | ◻ | ◻ | ◻ | ◻ |
| Government research institutions | ◻ | ◻ | ◻ | ◻ | ◻ |
| Insurance companies | ◻ | ◻ | ◻ | ◻ | ◻ |
| Pharmaceutical companies | ◻ | ◻ | ◻ | ◻ | ◻ |

**Data Sharing**

**During your participation in clinical trials, you have consented to the disclosure of your personal data to third parties in anonymized form. How concerned are you about the following possible consequences of sharing anonymous clinical trial data?**

|  | Strongly disagree | Disagree | Moderately agree | Agree | Strongly agree |
| --- | --- | --- | --- | --- | --- |
| My data could be used for marketing purposes instead of scientific purposes | ◻ | ◻ | ◻ | ◻ | ◻ |
| Companies or individuals could make great economic profit by using my data | ◻ | ◻ | ◻ | ◻ | ◻ |
| My data could be misused | ◻ | ◻ | ◻ | ◻ | ◻ |
| My data could be stolen | ◻ | ◻ | ◻ | ◻ | ◻ |
| My private data could be traceable with good computer knowledge | ◻ | ◻ | ◻ | ◻ | ◻ |
| By tracing my data, I could experience personal disadvantages | ◻ | ◻ | ◻ | ◻ | ◻ |

**Personality**

**To what extent do the following statements apply to you?**

|  | Strongly disagree | Disagree | Moderately agree | Agree | Strongly agree |
| --- | --- | --- | --- | --- | --- |
| I see myself as conventional, uncreative | ◻ | ◻ | ◻ | ◻ | ◻ |
| I see myself as dependable, self-disciplined | ◻ | ◻ | ◻ | ◻ | ◻ |
| I see myself as extraverted, enthusiastic | ◻ | ◻ | ◻ | ◻ | ◻ |
| I see myself as sympathetic, warm | ◻ | ◻ | ◻ | ◻ | ◻ |
| I see myself as anxious, easily upset | ◻ | ◻ | ◻ | ◻ | ◻ |
| I see myself as open to new experiences, complex | ◻ | ◻ | ◻ | ◻ | ◻ |
| I see myself as disorganised, careless | ◻ | ◻ | ◻ | ◻ | ◻ |
| I see myself as reserved, quiet | ◻ | ◻ | ◻ | ◻ | ◻ |
| I see myself as critical, quarrelsome | ◻ | ◻ | ◻ | ◻ | ◻ |
| I see myself as calm, emotionally stable | ◻ | ◻ | ◻ | ◻ | ◻ |

**Demographic Data**

**1) Gender**  ◻ Male ◻ Female

**2) Date of birth (YYYY)** _____________________________________

**3) Place of birth** _____________________________________

**4) Mother tongue** _____________________________________

**5) Marital status**

◻ Single

◻ Married

◻ Divorced

◻ Widowed

◻ Other

**6) Annual net income**

◻ <20,000€

◻ 21,000 - 40,000€

◻ 41,000 - 60,000€

◻ 61,000 – 80,000€

◻ 81,000 – 100,000€

◻ >100,000€
